# A Quick Reverse Transfection and Dual-Imaging Protocol for Simultaneous Viability and Oligonucleotide Delivery Assessment in 4T1 Cells

**DOI:** 10.3390/mps9050137

**Published:** 2026-09-20

**Authors:** Andrea González-Báez, Raúl Rangel-López, Gustavo Hernández-Vidal, Armando Trejo-Chávez, Luis Rodríguez-Tovar, Diana Zárate-Triviño, Diana Caballero-Hernandez, Rodolfo Nino Fong, Gerardo Méndez-Zamora, Diana Elisa Zamora-Avila

**Affiliations:** 1 Universidad Autónoma de Nuevo León, Facultad de Medicina Veterinaria y Zootecnia, San Nicolás de los Garza 66054, Nuevo Léon, Mexico; andrea.gonzalezbz@uanl.edu.mx (A.G.-B.); gustavo.hernandezvd@uanl.edu.mx (G.H.-V.); armando.trejochv@uanl.edu.mx (A.T.-C.); luis.rodrigueztv@uanl.edu.mx (L.R.-T.); 2 Universidad Autónoma de Nuevo León, Facultad de Ciencias Biológicas, San Nicolás de los Garza 66455, Nuevo Léon, Mexico; raul.rangellpz@uanl.edu.mx (R.R.-L.); diana.zaratetr@uanl.edu.mx (D.Z.-T.); diana.caballerohr@uanl.edu.mx (D.C.-H.); 3 Veterinary School, University of Long Island, New York, NY 11548, USA; rodolfo.ninofong@liu.edu; 4 Universidad Autónoma de Nuevo León, Facultad de Agronomía, San Nicolás de los Garza 66054, Nuevo Léon, Mexico; gerardo.mendezzm@uanl.edu.mx

**Keywords:** Xfect, polyethylenimine, chitosan, cell-associated uptake, complexation, 4T1 cells, gold nanoparticles, cell viability

## Abstract

The 4T1 cell line is a widely used model for triple-negative breast cancer (TNBC) research due to its high metastatic potential and resemblance to clinical progression. However, its biological characteristics often limit the efficiency and reproducibility of conventional nucleic acid delivery protocols. Here, we present an optimized non-viral delivery framework for efficient cell-associated uptake in 4T1 cells. Five carrier systems—Xfect RNA polymer (Xfect), polyethyleneimine (PEI), chitosan (CHI), and gold nanoparticle conjugates (AuPEI and AuCHI)—were systematically evaluated using both standard (adherent) and reverse (suspension) delivery approaches. A fluorescently labeled oligonucleotide (5′-TYE563) served as a reporter cargo at final concentrations of 5 nM and 10 nM, comparing complexation times of 15 s and 15 min. The proposed protocol introduces a rapid complexation strategy combined with reverse delivery, significantly reducing preparation time while enhancing cell-associated uptake. Under optimized conditions (10 nM, 15 s, reverse), Xfect achieved near-complete cellular uptake with minimal cytotoxicity. Crucially, the colocalization of dual-fluorescence signals enabled the simultaneous validation of metabolic viability and internalized cargo within a single field of view, bypassing the need for independent bulk cytotoxicity assays. This methodology provides a rapid, reproducible, and cost-effective framework to standardize early-stage delivery conditions for difficult-to-transfect cell lines such as 4T1.

## 1. Introduction

Breast cancer remains the most frequently diagnosed malignancy and the leading cause of cancer-related mortality among women worldwide, representing a major public health challenge [1,2]. According to recent estimates from the International Agency for Research on Cancer (IARC), approximately 2.31 million new breast cancer cases and 666,000 deaths occurred globally in 2022 [1,3]. Beyond its high mortality, analyses based on the Global Burden of Disease (GBD) 2021 study estimate that female breast cancer accounts for over 20 million Disability-Adjusted Life-Years (DALYs) worldwide. This DALY burden encompasses both Years of Life Lost (YLL) due to premature mortality and Years Lived with Disability (YLD), reflecting long-term functional impairment, treatment-related sequelae, and substantial socioeconomic impacts on patients and health systems [2,3].

In this context, gene-editing and nucleic acid-based technologies represent extremely valuable tools for investigating gene function and identifying therapeutic targets to improve patient prognosis; however, their translation relies heavily on efficient, safe, and reproducible delivery platforms [4,5].

Currently, several non-viral delivery methods are available, including physical and chemical approaches. Among the chemical reagents are polymers, polysaccharides, and cationic lipids, which facilitate the cellular uptake of oligonucleotides through charge interactions [6]. Non-viral agents have demonstrated safety in in vitro models, are relatively easy to use, and exhibit lower immunogenicity compared to viral vectors. These characteristics represent important advantages, making non-viral systems widely used as delivery tools [7].

Recent advances in DNA nanotechnology further illustrate the potential of nucleic acid structures as platforms for biomedical delivery. DNA strands can be programmably folded into well-defined nanostructures, such as DNA origami, and subsequently functionalized with molecular components to tailor their physicochemical and biological properties [8]. Such structural organization provides opportunities for incorporating or presenting functional molecules and may support applications in bioimaging and therapeutic delivery. In the context of breast cancer, DNA origami-based systems have been proposed as versatile platforms for drug loading and targeted delivery, while non-covalent modifications may improve their stability, cellular uptake, drug-loading capacity, and controlled release properties [9]. Although the present study does not investigate DNA origami or therapeutic drug delivery directly, these developments highlight the broader potential of nucleic acid-based structures and reinforce the importance of understanding and optimizing their interactions with target cells.

However, delivery efficiency and cytotoxicity depend on multiple factors, such as the cell line, incubation times, and experimental conditions, all of which can increase the cost and timeline of delivery-based projects [10,11]. Certain cell lines present greater challenges regarding cellular uptake compared to others, making it essential to identify and individualize optimal experimental conditions for each cell type. This is notably the case for the 4T1 cell line [12].

The 4T1 cell line is a tumor model of epithelial origin derived from a spontaneously arising mammary carcinoma in BALB/c mice. Although epithelial in origin, its biological behavior confers mixed epithelial/mesenchymal characteristics. The 4T1 model is characterized by the development of triple-negative tumors with metabolic features and metastatic patterns that closely resemble those observed in human patients, making it one of the most widely used preclinical models for the study of triple-negative breast cancer [13].

Murine 4T1 cells are recognized as being challenging to transfect, with highly variable delivery efficiencies reported across different studies. These inconsistencies have been attributed to several factors, including their rapid proliferation rate, growth characteristics, and unique features of their endocytic pathways and intracellular trafficking mechanisms [14]. Numerous delivery protocols for 4T1 cells have been described in the literature, most of which rely on conventional adherent conditions, incubation periods longer than 10 min, and relatively high reagent-to-oligonucleotide ratios [15,16]. Given the broad range of commercially available delivery reagents and the multiple parameters that influence cell-associated uptake efficiency in 4T1 cells, comparative evaluations of these systems represent valuable resources for optimizing experimental conditions, reducing costs, and improving reproducibility in studies employing the 4T1 model [17].

In the present study, a systematic evaluation of the cell-associated uptake efficiency of Xfect (Xfect), polyethyleneimine (PEI), chitosan (CHI), and gold nanoparticle conjugates with chitosan and polyethyleneimine (AuCHI and AuPEI) was performed using the fluorophore-labeled oligonucleotide 5′-TYE563^®^ (TYE563^®^) as a marker. A structured and reproducible methodology is presented with the aim of facilitating reagent selection and optimizing experimental conditions, thereby minimizing the costs and resources typically invested in the standardization of delivery protocols in projects that use 4T1 as an experimental model.

## 2. Materials and Methods

### 2.1. Cell Culture

Murine 4T1 (ATCC^®^ CRL-2539™) cells were maintained in Dulbecco’s Modified Eagle Medium (DMEM; Corning Incorporated, Corning, NY, USA) supplemented with 10% fetal bovine serum (FBS; Gibco, Thermo Fisher Scientific, Waltham, MA, USA), 100 U/mL penicillin and 100 µg/mL streptomycin (Corning Incorporated, Corning, NY, USA). Cell cultures were incubated (Sheldon Manufacturing, Inc., Cornelius, OR, USA) at 37 °C in a humidified atmosphere with 85% relative humidity and 5% CO_2_ until reaching 80–90% confluence.

### 2.2. Preparation of TYE563

A working solution of the TYE563^®^-labeled oligonucleotide (Integrated DNA Technologies, Inc., Coralville, IA, USA) was prepared at a concentration of 1000 nM in nuclease-free water (Integrated DNA Technologies, Inc., Coralville, IA, USA) to facilitate subsequent dilutions for the 5 nM and 10 nM working concentrations used in the assays. This fluorophore exhibits an excitation wavelength of 550 nm and an emission wavelength of 563 nm.

### 2.3. Preparation of Xfect

For all experiments, Xfect™ RNA Transfection Polymer (Takara Bio USA, Inc., San Jose, CA, USA) was used at a stock concentration of 100 µg/µL. The reagent was employed directly at a 1:1 volumetric ratio (*v*/*v*) relative to the volume of TYE563 working solution used in each assay.

### 2.4. Preparation of PEI

Polyethylenimine (PEI, branched/linear, 40 kDa; Sigma-Aldrich, St. Louis, MO, USA) solutions were prepared based on the molar concentration of its repeating monomeric units (MW = 43.07 g/mol), which provides a standardized measure of cationic amine density for nucleic acid complexation. Briefly, 0.323 g of PEI was dissolved in double-distilled water, and the final volume was adjusted to 50 mL to yield a 150 mM stock solution (in terms of monomeric units). A 10 mM working solution was subsequently prepared by diluting 66 µL of the stock solution in 934 µL of double-distilled water. This 10 mM working solution was used at a 1:1 volumetric ratio (*v*/*v*) relative to the volume of the TYE563 working solution applied in all cellular uptake assays (Figure 1).

### 2.5. Preparation of Low-Molecular-Weight Chitosan Solution

A 0.3% (*w*/*v*) working solution of low-molecular-weight chitosan (50–190 kDa; Sigma-Aldrich, St. Louis, MO, USA, according to the manufacturer’s specifications) was prepared in 0.1 M acetic acid. Briefly, the required volume of acetic acid was placed in a beaker, after which chitosan powder was added under continuous stirring for 24 h to ensure complete dissolution. The resulting solution was stored at 4 °C until further use (Figure 1).

### 2.6. Synthesis of Polyethylenimine-Reduced Gold Nanoparticles

Gold nanoparticles were synthesized using HAuCl_4_ as the metal precursor and polyethylenimine (PEI) as the reducing agent. A 2 mM HAuCl_4_ solution was prepared in double-distilled water and used in a 1:0.1 synthesis ratio with PEI.

Briefly, 5 mL of 2 mM HAuCl_4_ solution was placed in a beaker and heated in a water bath at 100 °C for 5 min (VWR). Subsequently, 500 µL of PEI at a concentration of 100 mM was added. The beaker was covered with aluminum foil and maintained under constant stirring and temperature for approximately 10 min.

Following this incubation period, the formation of gold nanoparticles was indicated by a visible color change to dark purple. After the color change was observed, the beaker was removed from the water bath and allowed to cool to room temperature. The resulting nanoparticle suspension was filtered through a 0.2 µm membrane filter and stored under refrigeration until further use (Figure 1).

### 2.7. Synthesis of Chitosan-Reduced Gold Nanoparticles

Gold nanoparticles were synthesized using HAuCl_4_ as the metal precursor and low-molecular-weight chitosan as the reducing agent. A 2 mM solution of HAuCl_4_ was prepared in double-distilled water, while chitosan was prepared at 1% (*w*/*v*) in 0.1 M acetic acid. The synthesis was performed using a 3:1 ratio of HAuCl_4_ to chitosan.

Briefly, 6 mL of the 2 mM HAuCl_4_ solution was placed in a beaker and heated in a water bath at 100 °C (VWR). Immediately afterward, 2 mL of 1% low-molecular-weight chitosan solution was added. The reaction mixture was maintained under these conditions for approximately 15 min.

Successful nanoparticle formation was indicated by a visible color change from yellow to a wine-red solution within the reaction time (Figure 1).

### 2.8. Characterization of Gold Nanoparticles

The synthesized gold nanoparticles functionalized with polyethyleneimine (AuPEI) and chitosan (AuCHI) were characterized in terms of hydrodynamic diameter and surface charge using dynamic light scattering (DLS) and zeta potential measurements, respectively. These parameters were selected because they are directly relevant to the colloidal behavior of the nanocarriers and their potential interaction with negatively charged oligonucleotides (Supplementary Materials).

### 2.9. Preparation of Delivery Vehicle Complexes

A key step in the complexation process between the delivery agents and the TYE563^®^ oligonucleotide was the initial dilution with 150 mM NaCl, which facilitated electrostatic self-assembly. In all cases, complexes were prepared using a 1:1 volumetric ratio between the delivery agent and TYE563^®^, regardless of the final TYE563^®^ concentration (5 nM or 10 nM).

Five delivery carriers were evaluated: polyethylenimine (PEI; Sigma-Aldrich, St. Louis, MO, USA), Xfect (Takara Bio USA, Inc., San Jose, CA, USA), chitosan (CHI), PEI–gold nanoparticle conjugates (AuPEI), and chitosan–gold nanoparticle conjugates (AuCHI). A fluorescently labeled oligonucleotide (5′-TYE563^®^; Integrated DNA Technologies, Inc., Coralville, IA, USA) was used as a reporter cargo to evaluate cell-associated uptake. Complexes were formed by mixing each delivery agent with 5′-TYE563^®^ at a 1:1 mass ratio, followed by volume adjustment with 150 mM NaCl. To evaluate the effect of complex assembly time, mixtures were incubated under medium-intensity vortex agitation for either 15 s or 15 min.

The detailed volumes, stock concentrations, and final working concentrations for the preparation of the delivery vehicle complexes are presented in Table 1.

### 2.10. 4T1 Delivery Protocols

Cells were subjected to two distinct delivery approaches, conventionally referred to in the literature as standard (adherent) and reverse (suspension) delivery approaches. In this study, these protocols were employed specifically to compare physical cellular uptake dynamics rather than functional gene expression.

### 2.11. Standard (Adherent) Delivery Approach

Cells were seeded in 24-well plates at a density of approximately 10,000 cells per well in 500 µL of complete DMEM supplemented with fetal bovine serum (FBS). After reaching 80–90% confluence, cell monolayers were rinsed with PBS. Subsequently, 300 µL of the preformed delivery vehicle complex was added, and the final volume was adjusted to 500 µL using fresh culture medium. Plates were incubated for 24 h at 37 °C. Complexes were assembled using two vortex-homogenization times (15 s or 15 min) prior to addition to the adherent monolayer.

### 2.12. Reverse (Suspension) Delivery Approach

When cultures reached 80–90% confluence in flasks, cells were washed with PBS and dissociated using 0.25% trypsin–EDTA (Gibco, Thermo Fisher Scientific, Waltham, MA, USA) for 5–10 min at 37 °C. The enzymatic reaction was neutralized with serum-supplemented medium. Suspended cells were centrifuged at 343× *g* for 5 min, and the resulting pellet was resuspended in fresh culture medium. Cell density and viability were determined using the Trypan Blue exclusion assay. An aliquot containing approximately 10,000 cells was transferred to each well of a 24-well plate already containing the pre-deposited delivery vehicle complexes (5 nM or 10 nM TYE563^®^). The mixture was gently homogenized, and 200 µL of culture medium was added to reach a final volume of 500 µL. As with the adherent approach, two complexation times were evaluated (15 s and 15 min), followed by a 24 h incubation period.

### 2.13. Cell Viability Assay

Cell viability was assessed using Calcein AM as a fluorescent viability marker. A 1 mM stock solution was prepared by resuspending 50 µg of Calcein AM powder in 50 µL of DMSO. A working solution was subsequently prepared by diluting the stock in PBS to a final concentration of 10 µM.

Following 24 h treatment with the delivery vehicle complexes in 24-well plates, the culture medium was removed, and cells were incubated with 10 µM Calcein AM in PBS for 30 min at 37 °C. The staining solution was then removed, and wells were washed twice with PBS to eliminate residual dye. Negative controls consisted of untreated cells without Calcein AM staining, whereas viability controls consisted of untreated cells stained with Calcein AM. Mean fluorescence intensity (MFI) of control wells, measured ImageJ version 1.54t (National Institutes of Health, USA; Java 1.8.0, represented 100% cell viability. Viability percentages for treated groups were calculated relative to the control group. Calcein AM exhibits excitation and emission wavelengths at 490 nm and 525 nm, respectively.

### 2.14. Cell-Associated Uptake Evaluation

To determine cell-associated uptake efficiency, each delivery carrier was complexed with 5′-TYE563^®^ at a 1:1 ratio. The objective was to trace the spatial localization of the fluorescent cargo and identify the system exhibiting the highest cell interaction. Dual-channel fluorescence microscopy was performed using an Olympus IX70 inverted microscope. Detection channels included green fluorescence for Calcein AM (viability, emission at 525 nm) and red fluorescence for TYE563^®^ (cargo uptake, excitation/emission at 550/563 nm). Spectral separation ensured simultaneous visualization without channel crosstalk.

### 2.15. Fluorescence Image Analysis and Quantification

Fluorescence images were processed using ImageJ version 1.54t (National Institutes of Health, USA; Java 1.8.0) under a standardized workflow. All images were acquired under identical exposure and gain settings. A uniform background subtraction was applied across all raw images. Linear adjustments were applied consistently to avoid green signal overflow into the red channel.

Quantification of Mean Fluorescence Intensity (MFI): Each image was divided into four equal non-overlapping quadrants analyzed independently. A minimum of three biological replicates per condition were evaluated (*n* = 12 measured fields per group). Quadrants devoid of cells were excluded.

Cell-Associated Uptake Analysis (Colocalization Approach): Uptake efficiency was determined by analyzing the colocalization of Calcein AM (green) and TYE563^®^ (red) signals. Single-channel images were merged. Positive cell-associated uptake events were identified strictly when both signals colocalized within the same cytosolic space, generating a merged yellow/orange signal.

Cell Counting and Normalization: The number of double-positive (Calcein AM^+^/TYE563^+^) cells was quantified manually or using semi-automated counting tools in ImageJ version 1.54t (National Institutes of Health, USA; Java 1.8.0). This value was normalized to the total number of viable cells, defined as Calcein AM–positive cells, according to the following equation:

$$Cell-associated\;uptake\left(\%\right)=\frac{Double-positive\;cells}{Total\;Calcein\;AM-positive\;cells}\times \;100$$

### 2.16. Methodological Considerations

This image-based quantification approach allows simultaneous evaluation of cell viability and nanoparticle-mediated delivery within the same sample, without disrupting the culture. Additionally, the use of quadrant-based sampling increases statistical robustness while maintaining spatial representativity.

Importantly, the spectral separation between Calcein AM (emission: ~525 nm) and TYE563 (excitation: ~550 nm) minimizes channel overlap, enabling reliable co-localization analysis.

### 2.17. Statistical Analysis

To determine the presence of statistically significant differences in cell viability, mean fluorescence intensity, and the percentage of interaction between delivery agents and 4T1 cells, data normality was first verified using the Shapiro–Wilk test. Subsequently, a one-way analysis of variance (ANOVA) was performed using the mean values of each experimental group, followed by Tukey’s post hoc test for multiple comparisons between treatments. All statistical analyses were conducted using GraphPad Prism (11.0.0) and a *p*-value of *p* < 0.05 was considered statistically significant.

## 3. Results

### 3.1. Physicochemical Characterization

Hydrodynamic diameter and surface charge (ζ-potential) of the functionalized gold nanoparticles were evaluated in aqueous dispersion via dynamic light scattering (DLS). AuPEI nanoparticles exhibited a mean hydrodynamic diameter of 83.17 ± 33.66 nm and a positive surface charge. AuCHI nanoparticles displayed a mean hydrodynamic diameter of 53.75 ± 27.06 nm and a ζ-potential of +31.1 mV. Both functionalized systems presented positive surface charge profiles, consistent with the cationic nature of the polymer coatings. Hydrodynamic parameters and surface charge values are summarized in Supplementary Materials.

### 3.2. Method Validation

Method validation incorporated MFI, cell-associated uptake (%), and relative cell viability (%). Murine 4T1 cells were exposed to delivery vehicle complexes assembled over 15 s or 15 min at 5 nM and 10 nM TYE563^®^ concentrations under standard and reverse delivery protocols. Following 24 h incubation, wells were washed with PBS and imaged. Single-stained controls established spectral de-coupling between Calcein AM (excitation/emission 495/515 nm) and TYE563^®^ (excitation/emission 549/563 nm), preventing false-positive colocalization. Quantification was performed across four representative fields per replicate using ImageJ. version 1.54t (National Institutes of Health, USA; Java 1.8.0).

### 3.3. Standard Delivery Approach

Under the standard adherent protocol at 5 nM TYE563^®^ and 15 s complexation, CHI and Xfect yielded the highest performance in both MFI (8.67 ± 1.2 and 12.76 ± 1.5, respectively) and cell-associated uptake (38.00% ± 3.2% and 58.67% ± 4.1%, respectively). CHI preserved cell viability near 100%. Extending complexation time to 15 min significantly reduced uptake below 40% across all agents, with CHI and Xfect achieving 29.40% and 30.10% uptake, respectively.

At 10 nM and 15 s complexation, CHI, AuPEI, and AuCHI displayed high MFI values (13.27, 9.00, and 14.30, respectively); however, Xfect provided superior cell viability (89.70%). Conversely, free PEI, AuPEI, and AuCHI significantly compromised cell viability at this concentration (62.20%, 55.40%, and 55.30%, respectively). Extending complexation to 15 min allowed Xfect to maintain higher viability, whereas CHI increased uptake at the expense of reducing viability below 80% (Figure 2).

### 3.4. Reverse (Suspension) Transfection Technique

The reverse delivery approach significantly enhanced uptake performance. At 5 nM and 15 s, Xfect, AuPEI, and AuCHI reached cell-associated uptake values of 60.34%, 72.86%, and 62.50%, respectively. AuPEI yielded the highest uptake despite exhibiting cytotoxicity (65.52% viability). At 15 min complexation in suspension, AuPEI (97.64%) and AuCHI (89.03%) nearly doubled the uptake efficiency of Xfect (54.36%). Nanoparticle-based agents showed reduced cytotoxicity with longer assembly times under suspension conditions (88.00%, 93.00%, and 100.00% viability).

At 10 nM and 15 s, Xfect achieved 94.47% cell-associated uptake without significant cytotoxicity relative to control. At 15 min, Xfect maintained 68.74% uptake with 100% viability, whereas free PEI showed minimal efficacy in both MFI (3.24) and uptake (1.89%) (Figure 3).

The reverse protocol enhanced MFI and uptake efficiency, likely due to the increased available cell surface area during suspension interaction compared to monolayer growth. Operational parameters are summarized in Table 2.

### 3.5. Limitations

While the optimized protocol presented here provides a reproducible framework for evaluating cell-associated uptake, the following limitations should be considered:

- Cell Line Specificity: Parameters were optimized specifically for 4T1 cells. Extension to other cell types requires individual calibration of reagent-to-cell ratios.
- Initial Cell Density: Reverse delivery relies strictly on initial suspension concentration (10,000 cells/well); variations may alter uptake and viability.
- Reporter Scope: The TYE563 oligonucleotide acts solely as a physical indicator of uptake. It lacks biological activity and does not assess downstream gene modulation.
- Fluorophore Stability: TYE563 is photosensitive. Exposure times must be strictly controlled to prevent photobleaching.
- Spectral Overlap: Viability and secondary stains must be selected with non-overlapping emission profiles relative to TYE563 (red spectrum).
- Complex Formation: Complex preparation in 150 mM NaCl prior to cell addition is mandatory to prevent uncoordinated aggregation during reverse seeding

## 4. Discussion

Breast cancer research heavily relies on the 4T1 murine model due to its highly metastatic, triple-negative profile [4]. However, its invasive nature renders it notoriously difficult to transfect using standard in vitro techniques [13]. This marked resistance is deeply rooted in the intrinsic biology of the model. Specifically, the refractiveness of the murine mammary adenocarcinoma 4T1 cell line to conventional non-viral transfection can be attributed to a combination of distinct extracellular, metabolic, and intracellular barriers. Extracellularly, their highly metastatic nature dictates a dense, unique surface profile—characterized by CD44 overexpression and the absence of human TFR1 receptors—that creates steric hindrance and restricts non-specific electrostatic interactions with chemical vectors. This shifts uptake toward specific receptor-mediated routes, active transport, and macropinocytosis, which are fueled by robust ATP-generating metabolic flexibility [18,19,20]. Transcriptomically, their rapid duplication rate, driven by Top2a and Mki67 overexpression, exponentially dilutes transfected genetic material among daughter cells, while Trp53 mutations increase susceptibility to reagent-induced toxicity [8]. Intracellularly, 4T1 cells exhibit hyperactive vesicular trafficking and ultra-accelerated lysosomal acidification kinetics driven by high V-ATPase pump density. This rapidly lowers vesicular pH (<5.0) and leads to severe endolysosomal entrapment, degrading transfection complexes before they can leverage the “proton-sponge” effect for endosomal escape [21,22]. Finally, species-specific differences amplify these barriers; unlike highly attenuated human reference lines (e.g., HEK293) that lack a functional cytosolic DNA-sensing apparatus, 4T1 cells maintain a competent, inducible cGAS-STING signaling axis. Upon encountering foreign nucleic acids, this pathway triggers downstream TBK1/IRF3 phosphorylation, type I interferon responses, and autophagic clearance protocols [23,24]. Collectively, this multi-layered defense mechanism—spanning restricted membrane binding, swift lysosomal sequestration, and active intracellular sensing—accounts for the suboptimal delivery outcomes observed in this specific murine model.

To address these delivery hurdles, we evaluated a rapid complexation strategy using an ultrashort 15 s mixing protocol. Comparative results indicate that the 15 s complexation step significantly outperforms the traditional 15 min incubation period, likely owing to the rapid kinetics of electrostatic self-assembly and the highly dynamic, non-equilibrium nature of polymer–oligonucleotide complex formation. Biophysical studies using time-resolved systems indicate that electrostatic attraction between cationic vectors and the negatively charged phosphate backbone of nucleic acids occurs rapidly upon mixing, suggesting that prolonged incubation may not be necessary for initial complex formation [25]. In this context, rapid vortexing during the initial 15 s complexation period promotes efficient and homogeneous contact between the delivery polymers and the oligonucleotide, favoring rapid electrostatic association before extensive polymer rearrangement or particle growth can occur. The short mixing period therefore helps capture the complexes in a kinetically trapped state, maintaining favorable colloidal properties and limiting secondary aggregation.

This kinetic control is particularly relevant for chitosan-based complexes. Because chitosan is a polycation whose protonated amino groups interact electrostatically with the negatively charged phosphate backbone of nucleic acids, prolonged incubation may allow progressive interparticle association, polymer chain rearrangement, and bridging between preformed complexes. Such processes can increase particle size, promote polydispersity, and ultimately lead to colloidal instability or precipitation. Rapid vortexing followed by immediate use of the complexes minimizes these time-dependent rearrangements by favoring the formation of smaller, more uniformly dispersed complexes before extensive aggregation occurs. Thus, the 15 s protocol preserves a favorable balance between rapid electrostatic trapping and the avoidance of subsequent thermodynamic aggregation, which is crucial for maintaining the dispersion and availability of chitosan-containing delivery systems [26].

By evaluating a 15 s window against a standard 15 min period, our intention was to examine distinct kinetic stages of complex assembly. Experimental data confirmed that restricting incubation to 15 s was associated with improved cell-associated uptake, whereas prolonged incubation was associated with reduced delivery performance. These findings support the use of a rapid complexation strategy as a practical approach for minimizing unnecessary incubation time and limiting colloidal growth prior to cellular exposure [26].

Traditionally, cells are transfected when already adhered to the culture vessel, limiting the surface area exposed to delivery agents [10]. In contrast, the reverse delivery approach allows cells to interact with delivery vehicle complexes while still in suspension. This method simplifies the workflow, reduces hands-on labor, and enables simultaneous cell seeding and transfection [27]. In our evaluations, the reverse technique yielded higher mean fluorescence intensity (MFI) and higher overall cell-associated uptake percentages compared to the standard technique.

We observed critical variations that closely align with the recent literature [28]. Although polymers are valuable tools for cellular transfection, the wide commercial variety demands rigorous evaluation of their interaction with cells, as their physicochemical properties dictate the delicate balance between intracellular delivery efficiency and cytotoxicity. Polyethylenimine (PEI) is widely used in non-viral transfection owing to its high efficiency; however, its high ionic charge density often causes significant cytotoxicity [29]. This was clearly reflected in our results, where PEI-based agents severely impaired the viability of 4T1 cells. Nevertheless, complexing PEI with gold nanoparticles mitigated its cytotoxic effect while enhancing the cell-associated uptake percentage. Chitosan (CHI), on the other hand, is a linear biopolymer renowned for its high biocompatibility, biodegradability, and non-toxicity [29]. In our experiments, chitosan successfully preserved cell viability. Although chitosan appears to increase MFI, evaluating the cell-associated uptake percentage (yellow fluorescence signal) reveals that it underperforms relative to PEI. This discrepancy is likely attributable to the large molecular size of chitosan, which creates biological hurdles to overcoming endosomal escape barriers, as documented in the literature [28,29].

To overcome these limitations through nanostructuring, PEI and chitosan were utilized as functional coatings, as both polymers possess a high intrinsic density of positive charges that favor electrostatic complexation with negatively charged nucleic acids [30,31]. For AuPEI nanoparticles, DLS analysis yielded a hydrodynamic diameter of 83.17 ± 33.66 nm. The nanoparticles exhibited a positive surface charge, consistent with the presence of protonated amino groups associated with PEI. The observed hydrodynamic size is within the range reported for comparable PEI-functionalized gold nanoparticles. Cho et al. [32] demonstrated that the synthetic route used for the preparation of PEI-functionalized gold nanoparticles can substantially influence their physicochemical properties, including particle size and surface charge. Their study reported AuPEI systems with particle sizes in the nanometer range and positive zeta potentials, supporting the suitability of PEI-mediated approaches for generating cationic gold nanocarriers.

AuCHI nanoparticles exhibited a hydrodynamic diameter of 53.75 ± 27.06 nm and a positive zeta potential of +31.1 mV. The positive surface charge is consistent with the protonation of chitosan amino groups under the acidic conditions used for preparation and is relevant to the electrostatic association of the nanoparticles with negatively charged nucleic acids. These results are consistent with previous reports describing the preparation and characterization of chitosan-capped or chitosan-stabilized gold nanoparticles. Mohan et al. [33] reported chitosan-capped gold nanoparticles with hydrodynamic sizes that varied according to the chitosan concentration, demonstrating that the polymer concentration can influence nanoparticle size, polydispersity, and surface charge. Similarly, Potara et al. [34] demonstrated that chitosan can act as both a reducing and stabilizing agent during gold nanoparticle synthesis and showed, using transmission electron microscopy (TEM) and complementary physicochemical techniques, that synthesis conditions can influence the size and morphology of chitosan-associated gold nanoparticles. These physicochemical attributes—hydrodynamic diameters below 85 nm and strong positive surface charges—are well-established drivers for enhancing electrostatically mediated associations with the negatively charged outer plasma membrane, thereby improving cellular uptake relative to their free polymer counterparts [35].

It is important to distinguish the measurements obtained by DLS from those obtained by electron microscopy. DLS determines the hydrodynamic diameter of particles dispersed in liquid, which reflects not only the inorganic nanoparticle core but also the associated hydration layer and surface-bound molecules. In contrast, TEM or scanning electron microscopy (SEM) provides information regarding the physical dimensions and morphology of the electron-dense nanoparticles, generally under dried or vacuum conditions. Consequently, DLS-derived hydrodynamic diameters may differ from particle dimensions determined by TEM or SEM without necessarily indicating differences in nanoparticle synthesis or quality.

The positive surface charge observed for both AuPEI and AuCHI is particularly relevant to their intended application as oligonucleotide delivery vehicles. Electrostatic interactions between cationic surface groups and the negatively charged phosphate backbone of nucleic acids can promote nanoparticle–oligonucleotide association and contribute to cellular uptake. Thus, the DLS and zeta potential measurements provide relevant physicochemical information for interpreting the subsequent evaluation of oligonucleotide-associated cellular uptake in 4T1 cells.

TEM or SEM analysis was not performed in the present study and, therefore, the primary particle morphology, physical dimensions, and aggregation state were not directly determined by electron microscopy. This represents a limitation of the current physicochemical characterization. Nevertheless, the DLS and zeta potential measurements provide parameters directly relevant to the methodological objective of this study, which was to evaluate and compare different oligonucleotide delivery vehicles under standardized conditions. Future studies incorporating TEM or SEM, together with complementary techniques such as polydispersity analysis, elemental characterization, and assessment of colloidal stability, will provide a more comprehensive physicochemical characterization of the synthesized AuPEI and AuCHI nanoparticles.

Although free chitosan yielded high MFI values and apparent interaction with 4T1 cells, detailed fluorescence image analysis revealed that a substantial proportion of the TYE563 signal remained localized in the extracellular space, as evidenced by limited colocalization between Calcein AM and TYE563. This observation indicates that free chitosan—likely due to its lower solubility at physiological pH (~7.4) and tendency to form large colloidal aggregates—yields bulky complexes that adhere to the cell surface without undergoing efficient intracellular internalization [36,37]. This physicochemical limitation explains why free chitosan exhibited elevated MFI levels alongside lower percentages of double-positive cells compared to AuCHI and the commercial transfection control (Xfect). The integration of the gold nanoparticle core likely provides a rigid structural template that restricts polymer chain flexibility, reduces steric hindrance, and optimizes nanoparticle geometry for cell–carrier interactions [38]. Conjugating these polymers with gold nanoparticles (AuPEI, AuCHI) to anchor them to a metallic core minimizes detrimental direct interactions with the cell membrane [39]. Interestingly, the prolonged surface association observed for free chitosan complexes does not necessarily represent a complete drawback [29]; such surface anchoring could favor sustained nucleic acid release over extended time frames. In rapidly proliferating models like 4T1 cells, continuous exposure to released genetic cargo might enhance cumulative uptake over time [40]. Nevertheless, further kinetic studies evaluating long-term cell-associated uptake dynamics and downstream functional gene expression are warranted.

The Xfect polymer demonstrated the best experimental balance in our study, achieving almost complete cellular uptake without affecting viability. Modern non-viral delivery systems like Xfect have been specifically optimized to possess a low cytotoxicity profile while maintaining high transfection rates, making them ideal for gene delivery in difficult-to-transfect cancer cell models [7]. Notably, the commercial reference carrier Xfect demonstrated the most favorable balance in our experimental conditions, achieving near-complete cellular uptake without compromising metabolic viability. Although our previous comparative data highlight polymers with a favorable balance of cell-associated uptake and cell viability, future studies must contrast these systems with agents operating under distinct physicochemical mechanisms. This is the case for cationic lipids, which have proven to be reliable and safe tools for the transfection of various cell lines [41]. Contrasting the delivery capacity of systems with divergent molecular natures will comprehensively establish which physicochemical platform offers the optimal risk–benefit ratio for transfecting complex in vitro models, such as the 4T1 cell line [42].

We observed a decline in cytotoxicity when extending the conjugation time for the gold nanoparticle complexes. Although existing reports indicate that bare gold nanoparticles compromise cell viability through surface-driven reactivity, our findings suggest that longer assembly times with RNA shift this dynamic [43]. Prolonged incubation likely yields a higher packing density of the nucleic acids on the nanoparticle core. By maximizing RNA binding, this effectively shields the reactive metal interface, preventing direct, damaging interactions with the cell membrane and ultimately mitigating the overall cytotoxic effect [44,45].

Another key advantage of the optimized reverse delivery approach presented in this study is the ability to simultaneously determine both cell viability and cell-associated uptake efficiency within a single fluorescence microscopy field of view [46]. Calcein AM plays a central role here, as this acetoxymethyl ester is cell-permeable and non-fluorescent in its native state. However, once inside a living cell, intracellular esterases hydrolyze it into calcein, a fluorophore that emits an intense green signal, confirming that the recorded green fluorescence originates from metabolically active, viable cells [47]. Unlike endpoint metabolic assays that produce formazan—which require solvent-mediated cell lysis to dissolve crystalline precipitates—the present strategy enables continuous evaluation of living, non-fixed specimens. Consequently, sample destruction is avoided, preserving biological integrity throughout the monitoring period [48].

The detection of the labeled oligonucleotide (conjugated with the red fluorophore TYE563) allows direct observation and quantification of successfully transfected cells. Simultaneous dual-channel visualization enables confirmation of the spatial localization of the delivered genetic material. The overlay of emission filters corresponding to the red (TYE563) and green (calcein) signals produces a yellow colocalization signal, indicating that both the oligonucleotide and calcein are present within a viable cell. This strategy may prove equally useful for assays targeting other cellular compartments, such as the nucleus, where fluorescent reporters in the red or blue spectral ranges require specific spatial localization [46].

Beyond identifying successful internalization events, a fundamental advantage of this dual-color imaging logic is its capacity to simultaneously evaluate cellular viability and discriminate non-specific signals in a single step. By relying on the colocalization of Calcein AM (green) and TYE563 (red), successful uptake into metabolically active cells is unambiguously identified by a merged yellow/orange signal. Rather than being mere imaging artifacts, isolated single-color signals observed in the extracellular space or associated with non-viable cells provide valuable mechanistic insights. For instance, intense extracellular red puncta indicate uninternalized nucleic acid complexes that remain associated with the culture substrate. This phenomenon was observed predominantly with chitosan formulations, consistent with the relatively large particle size and limited solubility of chitosan under physiological culture conditions. Similarly, red-only signals spatially associated with cellular morphology allow for the identification of cells that interacted with the delivery vehicle but subsequently lost metabolic viability. Conversely, isolated extracellular green fluorescence likely stems from released calcein following cell membrane damage or residual cellular debris, as Calcein AM strictly requires intracellular esterase hydrolysis to fluoresce. By distinguishing these discrete populations, the dual-color method successfully discriminates true positive events from extracellular complexes and cell debris. Furthermore, it is important to consider the intrinsic biological characteristics of the model; for example, the 4T1 cell line naturally tends to grow in densely packed clusters. While this growth pattern can make precise delineation of individual cell boundaries challenging under standard wide-field microscopy, the robust yellow colocalization signals consistently map to morphologically identifiable cellular regions.

While traditional nucleic acid delivery assays frequently rely on nuclear counterstains to provide spatial reference, such markers were intentionally omitted in this protocol to preserve the physiological integrity of the cells and maintain the single-step nature of the assay. Live-cell nuclear dyes, such as Hoechst 33342, require ultraviolet (UV) excitation, which rapidly induces oxidative stress and phototoxicity. This UV-induced stress could confound the true metabolic viability measurements provided by Calcein AM. By relying exclusively on less energetic visible light, this non-destructive dual-color approach maintains the native metabolic state of the cells. A significant advantage of this minimally invasive strategy is that, following imaging, cells remain fully viable and can be safely returned to culture for subsequent longitudinal analyses to evaluate long-term delivery effects [49,50]. Furthermore, because Calcein AM distributes evenly throughout the cytosol of metabolically active cells, it effectively outlines the entire cellular volume, serving as a functional morphological marker. When combined with bright-field microscopy, this allows for a robust spatial assessment of TYE563 overlap with viable cells. However, acknowledging the inherent limitations of wide-field fluorescence microscopy in the absence of precise nuclear or membrane counterstaining, this optical configuration cannot unequivocally distinguish cargo internalized deep within the cytoplasm from complexes strongly adhered to the plasma membrane. Therefore, positive colocalization events in this methodology are conservatively defined as “cell-associated uptake” rather than definitive intracellular delivery. For future applications demanding precise subcellular localization, such as plasmid DNA transfection, this protocol should be supplemented with appropriate live-cell spatial counterstains and confocal optical sectioning.

This dual-imaging approach bypasses the need for prior, independent cytotoxicity assays like tetrazolium-based colorimetric tests (e.g., MTT), which are often time-consuming and prone to interference artifacts. By assessing the presence of fluorescent calcein alongside cell-associated uptake via TYE563 fluorescence, our protocol functions as a real-time assay for both viability and cell-associated uptake efficiency [51]. In this study, cellular uptake efficiencies and cell viability were quantified using automated analysis of fluorescence micrographs across 240 standardized images (5 treatments, 2 concentrations, and 2 conjugation times evaluated in triplicate, with 4 independent fields captured per replicate). While this approach provided crucial spatial and metabolic confirmation of cell-associated uptake, we fully recognize the importance of flow cytometry for high-throughput statistical evaluation; therefore, we plan to utilize flow cytometry in future studies to rigorously validate these results.

In terms of cargo compatibility, the protocol optimized in this study was specifically validated using small single- and double-stranded oligonucleotides (e.g., siRNA). While the single-step dual-color screening logic remains conceptually applicable to other nucleic acid delivery systems, larger cargos such as plasmid DNA or mRNA exhibit distinct molecular sizes and charge distributions. Therefore, scaling this protocol for macromolecular cargos requires specific re-optimization of the vehicle-to-cargo ratio to maintain complex stability and avoid excessive vehicle concentration. The parameters presented here serve as a validated benchmark specifically for small regulatory RNA and oligonucleotide delivery systems [46].

It is important to acknowledge that the experimental findings of the present study primarily characterize cellular interaction and cell-associated uptake rather than functional transfection. Our evaluations focused on quantifying cellular uptake and localization, leaving downstream biological outcomes—such as gene expression kinetics or targeted gene silencing—outside the immediate scope of this work. Consequently, observed cell-associated uptake efficiencies should not be directly interpreted as functional gene modulation. Future investigations will incorporate functional assays to determine the biological efficacy and gene regulation performance of nucleic acids delivered within these polymeric and gold nanoparticle-based platforms.

This protocol can be successfully adapted to simultaneously evaluate the efficiency and toxicity of other vectors, provided their emission spectra fall within the blue or red ranges, thereby reserving the green spectrum for calcein-mediated viability confirmation [46]. Overall, this methodology represents a rapid, streamlined tool that reduces experimental steps while accurately validating both cell-associated uptake and the biocompatibility of polymers or nanoparticles in difficult-to-transfect models like the 4T1 cell line. Future research should focus on adapting this optimized protocol for other notoriously recalcitrant cell lines and evaluating its scalability for high-throughput screening applications.

## 5. Conclusions

A 15 s complexation step combined with a reverse delivery approach significantly improves cell-associated oligonucleotide uptake in 4T1 cells. The non-destructive, single-step dual-color imaging setup using Calcein AM and 5′-TYE563^®^ enables real-time, simultaneous quantification of cell viability and cargo uptake within a single field of view, bypassing redundant bulk cytotoxicity assays.

Overall, this method provides a reproducible and flexible framework for in vitro models using RNAi. Rather than serving as a universal solution, this protocol offers a reliable baseline and starting point for standardizing delivery conditions across other RNAi and nucleic acid carrier systems or reporter constructs—such as plasmids encoding fluorescent proteins—provided appropriate emission spectra are selected. By reducing preparation time, bypassing redundant viability assays, and optimizing reagent usage, this strategy represents a practical and systematic tool to guide the standardization of molecular delivery studies in cancer research. Building upon these findings, future research will focus on expanding the physicochemical characterization of the optimized nanocomplexes, including colloidal stability, nucleic acid loading efficiency, and detailed structural morphology. Additionally, mechanistic studies investigating intracellular trafficking, endosomal escape, and downstream biological effects will be essential to validate functional delivery following the administration of biologically active nucleic acids. Finally, evaluating this rapid complexation protocol across other challenging, hard-to-transfect cell lines as well as physiologically relevant breast cancer models will further establish the versatility and translational potential of these delivery systems.

## Figures and Tables

**Figure 1:**
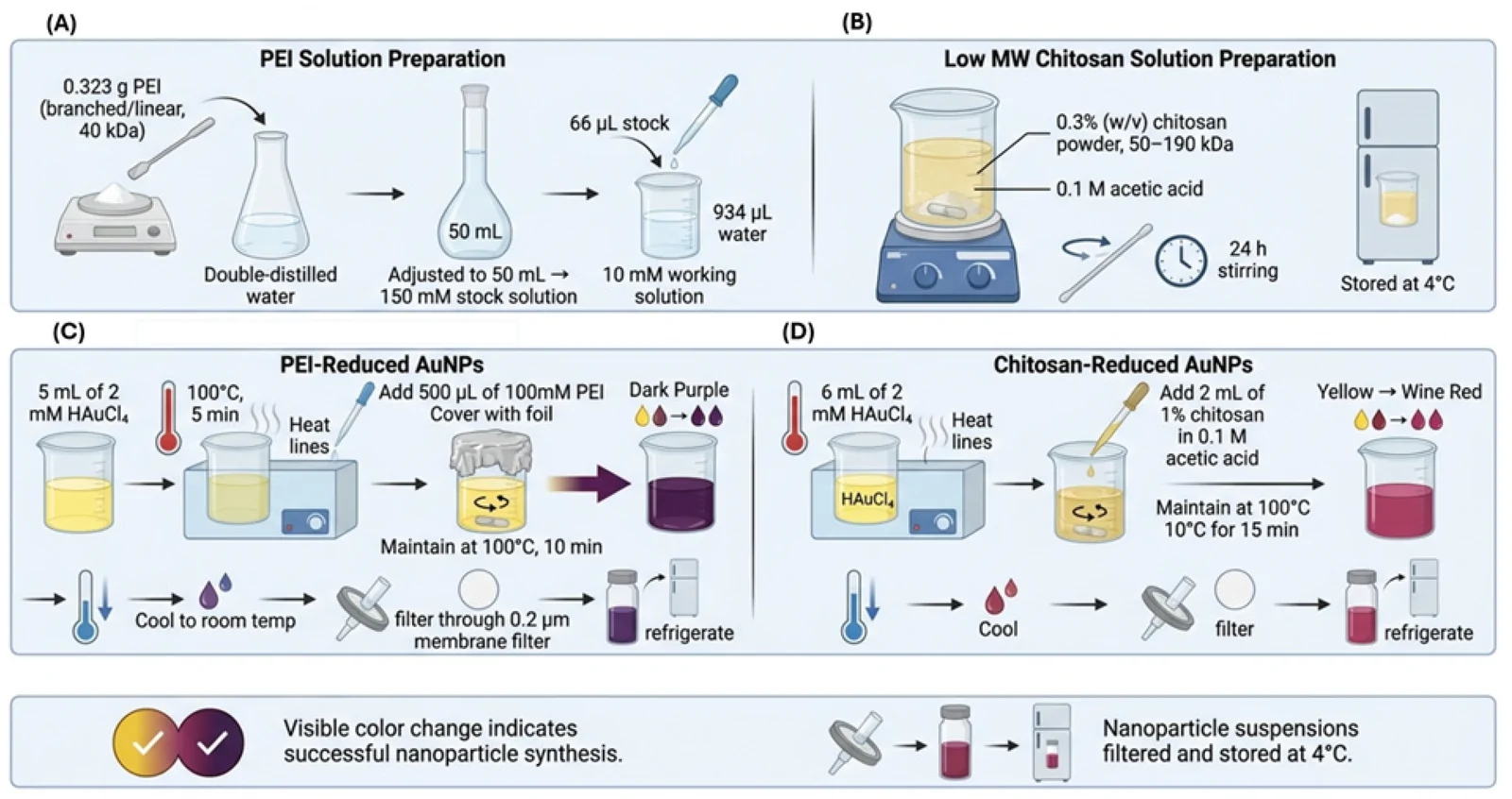
Schematic representation of the preparation of the non-viral delivery systems. (**A**) Preparation of polyethylenimine (PEI) working solution. (**B**) Preparation of low-molecular-weight chitosan (CHI) working solution. (**C**) Synthesis of PEI-reduced gold nanoparticles (AuPEI). (**D**) Synthesis of chitosan-reduced gold nanoparticles (AuCHI).

**Figure 2:**
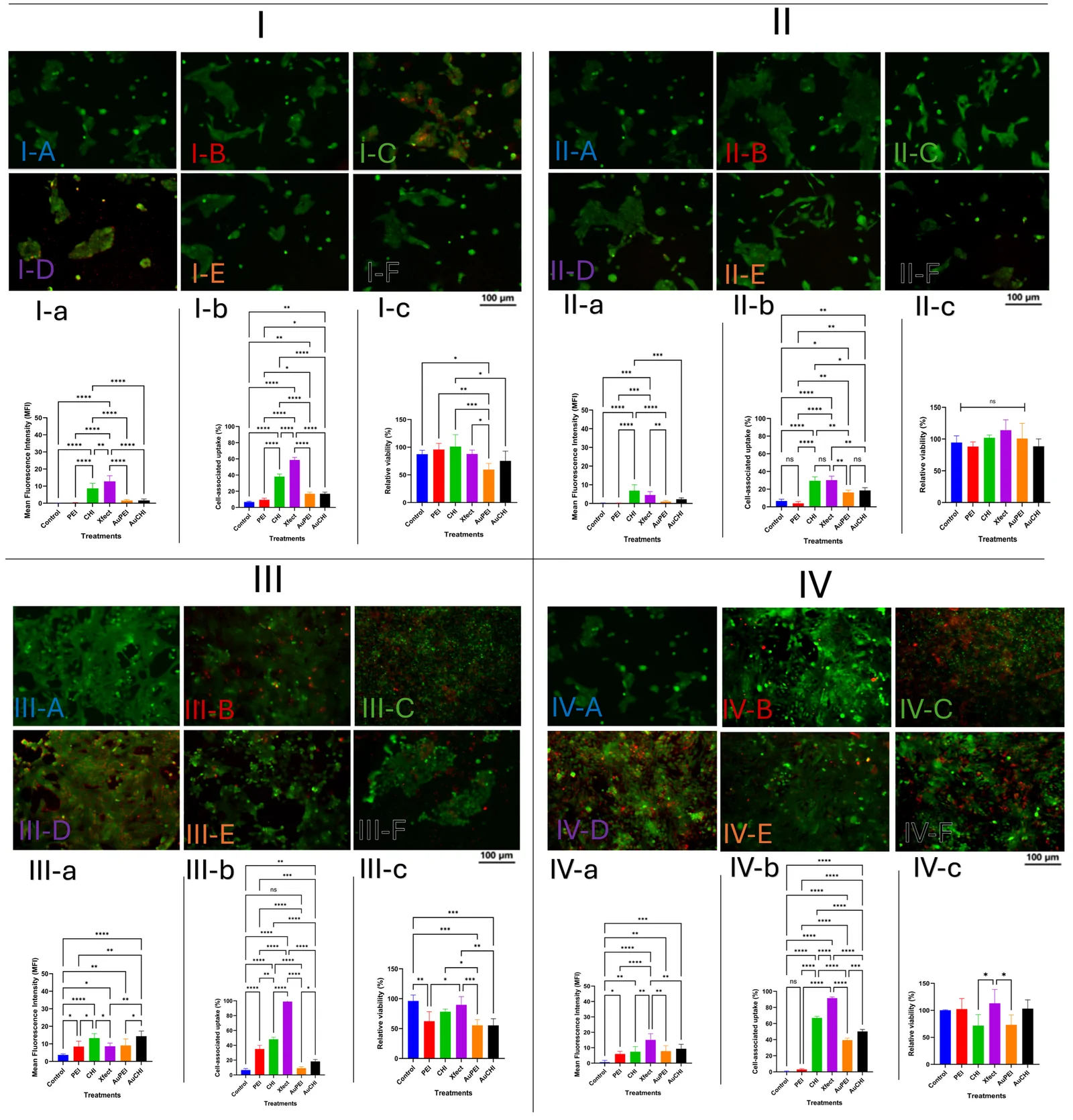
Quantitative evaluation of cell-associated uptake and cell viability using the standard (adherent) delivery approach in 4T1 cells. Representative micrographs (100 µm) and corresponding quantitative metrics for (**I**) 5 Nm conjugate incubated for 15 s; (**II**) 5 nM conjugate incubated for 15 min; (**III**) 10 nM conjugate incubated for 15 s; and (**IV**) 10 nM conjugate incubated for 15 min. Micrographs were acquired at 10× magnification. Treatments: (**I-A**–**IV-A**) Untreated Control (blue), (**I-B**–**IV-B**) PEI (red), (**I-C**–**IV-C**) CHI (green), (**I-D**–**IV-D**) Xfect (purple), (**I-E**–**IV-E**) AuPEI (orange), and (**I-F**–**IV-F**) AuCHI (black). Quantitative graphs: (**I-a**–**IV-a**) Mean Fluorescence Intensity (MFI) evaluated using TYE563; (**I-b**–**IV-b**) cell-associated uptake percentage (colocalization of Calcein AM and TYE563); and (**I-c**–**IV-c**) relative cell viability percentage assessed by Calcein AM fluorescence. Statistical Analysis and Sample Size: Data are expressed as mean ± standard deviation (SD) from *n* = 3 independent biological replicates (*n* = 12 analyzed fields per condition, representing 4 non-overlapping quadrants per well across 240 standardized images). Data normality was confirmed using the Shapiro–Wilk test. Statistical significance was determined by one-way analysis of variance (ANOVA) followed by Tukey’s post hoc test for multiple comparisons (* *p* < 0.05, ** *p* < 0.01, *** *p* < 0.001, **** *p* < 0.0001; ns = not significant).

**Figure 3:**
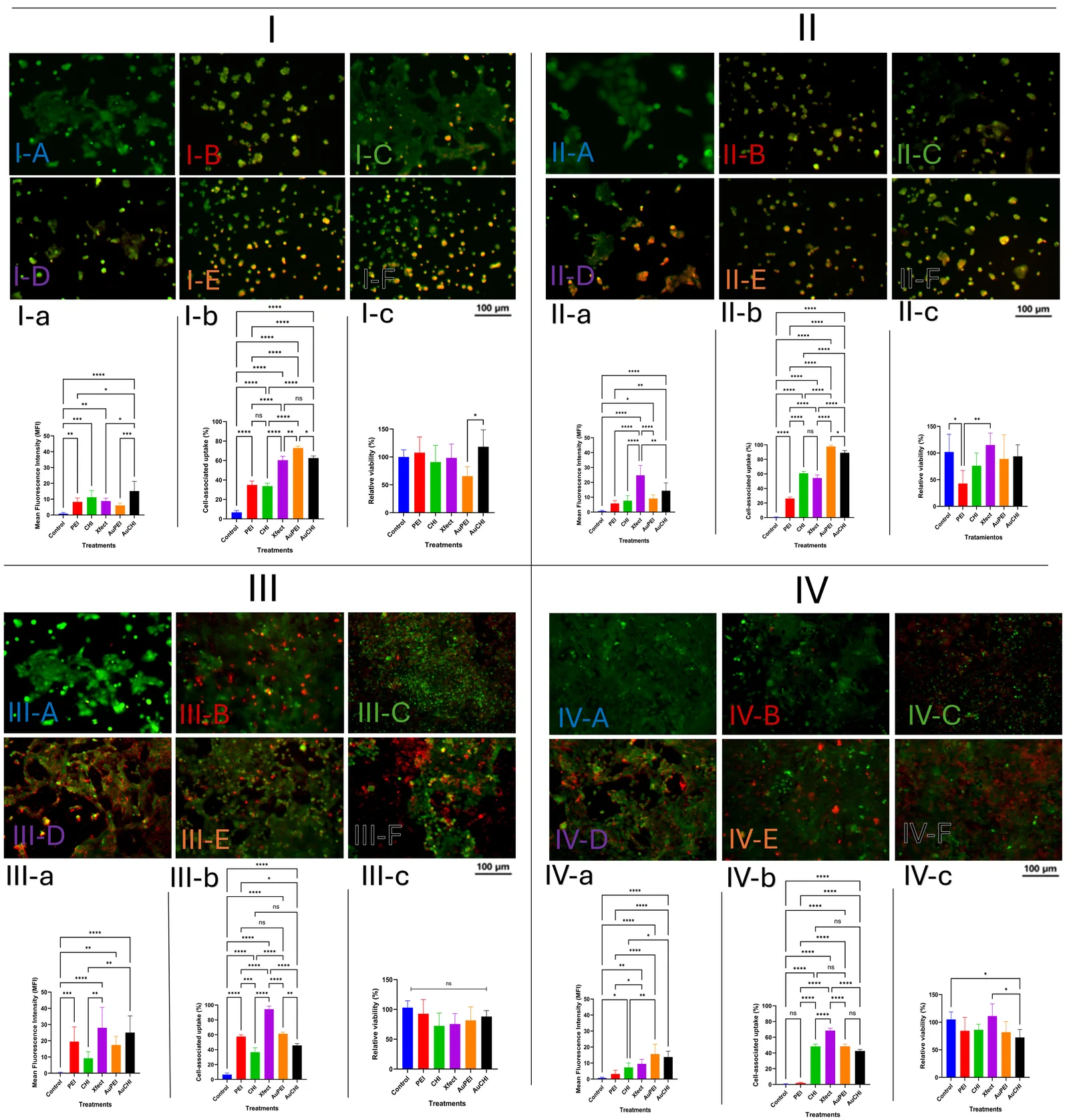
Quantitative evaluation of cell-associated uptake and cell viability using the reverse (suspension) delivery approach in 4T1 cells. Representative micrographs (100 µm) and corresponding quantitative metrics for (**I**) 5 nM conjugate incubated for 15 s; (**II**) 5 nM conjugate incubated for 15 min; (**III**) 10 nM conjugate incubated for 15 s; and (**IV**) 10 nM conjugate incubated for 15 min. Micrographs were acquired at 10× magnification. Treatments: (**I-A**–**IV-A**) Untreated Control (blue), (**I-B**–**IV-B**) PEI (red), (**I-C**–**IV-C**) CHI (green), (**I-D**–**IV-D**) Xfect (purple), (**I-E**–**IV-E**) AuPEI (orange), and (**I-F**–**IV-F**) AuCHI (black). Quantitative graphs: (**I-a**–**IV-a**) Mean Fluorescence Intensity (MFI) evaluated using TYE56; (**I-b**–**IV-b**) cell-associated uptake percentage (colocalization of Calcein AM and TYE563); and (**I-c**–**IV-c**) relative cell viability percentage assessed by Calcein AM fluorescence. Statistical Analysis and Sample Size: Data are expressed as mean ± standard deviation (SD) from *n* = 3 independent biological replicates (*n* = 12 analyzed fields per condition, representing 4 non-overlapping quadrants per well across 240 standardized images). Data normality was confirmed using the Shapiro–Wilk test. Statistical significance was determined by one-way analysis of variance (ANOVA) followed by Tukey’s post hoc test for multiple comparisons (* *p* < 0.05, ** *p* < 0.01, *** *p* < 0.001, **** *p* < 0.0001; ns = not significant).

**Table 1:** Detailed preparation of delivery vehicle complexes showing the volumes, stock concentrations, and final concentrations of each component. All complexes were prepared using a 1:1 volumetric ratio (*v*/*v*) between the delivery carrier and TYE563.

Delivery Agent	PEI (10 mM)	CHI (0.3% *p*/*v*)	Xfect (100 µg/µL)	AuPEI (2 mM/10 mM)	AuCHI (2 mM/0.3%)	TYE563 1000 nM	NaCl 150 mM	Culture Medium
5 nm	5 µL	5 µL	5 µL	5 µL	5 µL	5 µL	190 µL	300 µL
10 nm	10 µL	10 µL	10 µL	10 µL	10 µL	10 µL	180 µL	300 µL

**Table 2:** Key operational parameters.

Delivery Approach	Dose (nm)	Complexation Time	Best Performing Agent (>50% of Percentage of Cell-Associated Uptake)	Cell Viability	Key Observations
Standard	5	15 s	CHI/Xfect	~100% (CHI)	Best balance between efficiency and viability
Standard	5	15 min	None	Variable	Extended complexation reduces efficiency
Standard	10	15 s	Xfect	>90%	Optimal viability at higher dose
Standard	10	15 min	Xfect	~100%	Maintains viability but reduced efficiency
Reverse	5	15 s	AuPEI/Xfect	Reduced (AuPEI)	Higher efficiency with increased cytotoxicity
Reverse	5	15 min	AuPEI/AuCHI	Reduced	Nanoparticles enhance cell-associated uptake but affect viability
Reverse	10	15 s	Xfect	~100%	Optimal condition overall
Reverse	10	15 min	Xfect	~100%	Stable performance across conditions

## Data Availability

The original contributions presented in the study are included in the article/Supplementary Material; further inquiries can be directed to the corresponding author.

## Supplementary Materials

The following supporting information can be downloaded at https://www.mdpi.com/article/10.3390/mps9050137/s1, Gold nanoparticle characterization.

## Abbreviations

The following abbreviations are used in this manuscript:

AM	Acetoxymethylester
AuCHI	Gold nanoparticle conjugates with chitosan
AuPEI	Gold nanoparticle conjugates with polyethyleneimine
CHI	Chitosan
DLS	Dynamic light scattering
DMEM	Dulbecco’s Modified Eagle Medium
DMSO	Dimethyl sulfoxide
EDTA	Ethylenediaminetetraacetic acid
FBS	Fetal bovine serum
MFI	Mean Fluorescence Intensity
MTT	Tetrazolium-based colorimetric tests
PBS	Phosphate-Buffered Saline
PEI	Polyethyleneimine
TNBC	Triple-negative breast cancer
