# Supplementary material for: A Quick Reverse Transfection and Dual-Imaging Protocol for Simultaneous Viability and Oligonucleotide Delivery Assessment in 4T1 Cells
Source: Methods Protoc. 2026 Sep 20;9(5):137. doi: 10.3390/mps9050137 (PMC13638061; doi:10.3390/mps9050137)
Supplement: Supplementary file 1 [file mps-09-00137-s001.zip › mps-4437471-Supplementary.pdf]

Type of nanoparticles	Size	Z potential
AuPEI	83.17 (+/- 33.66)	27.9 (+/- 10.8)
AuCHI	53.75 (+/- 27.06)	31.1 (+/- 13.3)

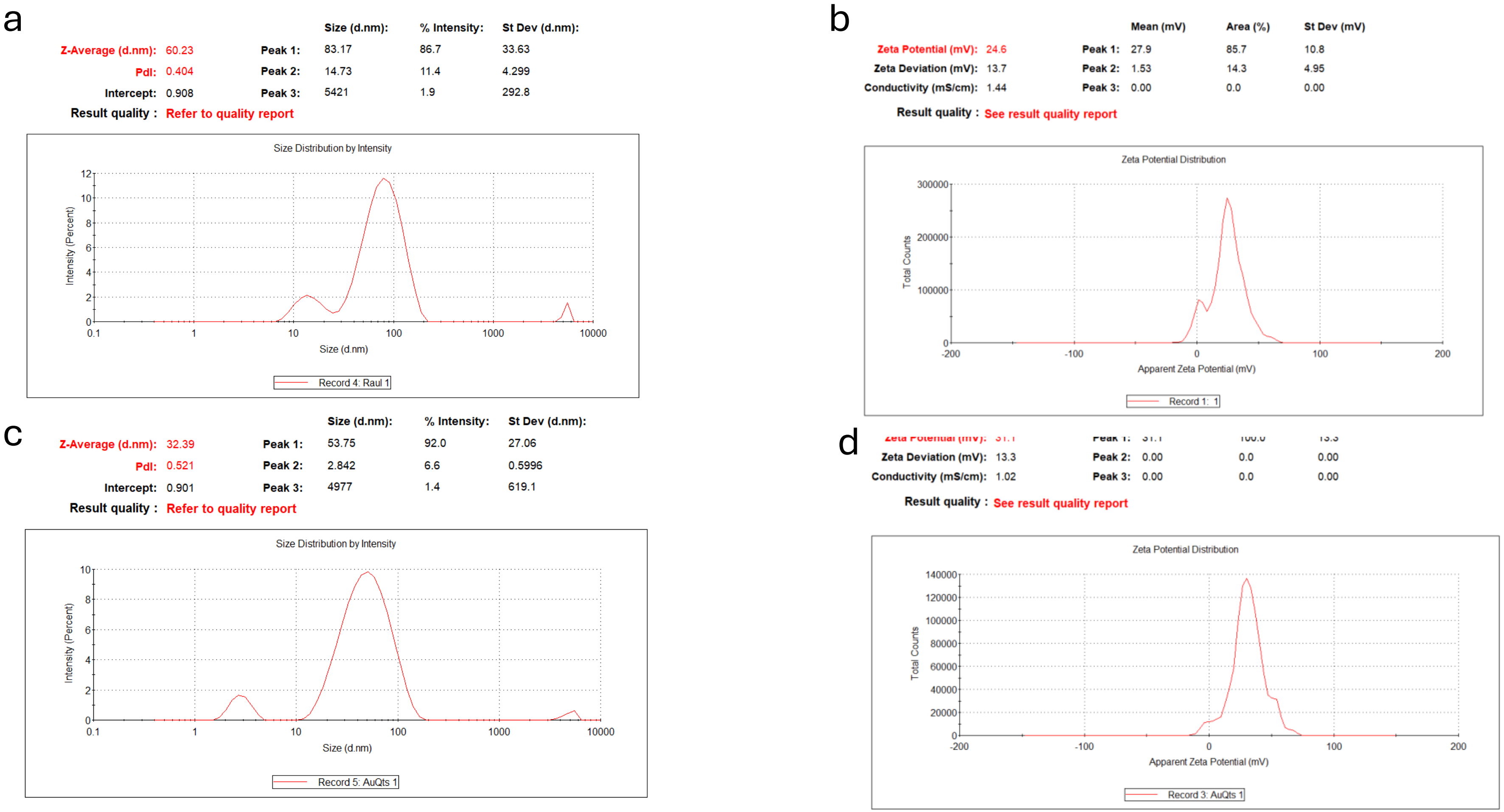

Gold nanoparticle characterization by dynamic light scattering (DLS) for size and surface charge (ζ-potential). The upper panel shows the size and surface charge characterization of AuPEI and AuCHI nanoparticles, respectively, performed using a Nanosizer NS90 instrument (Malvern Instruments, Malvern, UK).Panels (a) and (b) correspond to the average size and ζ-potential of AuPEI nanoparticles, respectively, whereas panels (c) and (d) correspond to those of AuCHI nanoparticles.
